# Integrated reporting disclosure scoring dataset for Malaysian Shariah-compliant public listed companies (2020–2023)

**DOI:** 10.1016/j.dib.2026.112815

**Published:** 2026-05-05

**Authors:** Wai Kee Ho, Nampuna Dolok Gultom, Ferdinand Akthar Khan Gul, Susela Devi K. Suppiah, Khakan Najaf, Emily Kok, Eng Sze Phuah

**Affiliations:** a Sunway University, No. 5, Jalan Universiti, Bandar Sunway, 47500 Selangor Darul Ehsan, Malaysia; b University of the Sunshine Coast, 90 Sippy Downs Drive, Sippy Downs QLD 4556, Australia; c Universiti Selangor (UNISEL) Malaysia, Jalan Zirkon A7/A, Seksyen 7 40000 Shah Alam Selangor, Malaysia; d Canadian University Dubai, City Walk, Al Wasl, Dubai, United Arab Emirates

**Keywords:** Integrated reporting, Disclosure scoring, Content analysis, Corporate reporting, Shariah-compliant firms, Malaysia

## Abstract

This article presents a dataset on Integrated Reporting disclosure scores for Malaysian Shariah-compliant public listed companies covering the period 2020–2023. The dataset was constructed through systematic manual content analysis of corporate annual reports and integrated reports obtained from publicly available company disclosures. A structured coding framework consisting of thirteen integrated reporting components derived from the International Integrated Reporting Council (IIRC) Framework was applied to evaluate disclosure practices.

Each component was assessed using an ordinal scoring scale ranging from 0 to 5 to capture the depth and quality of disclosure. The dataset contains firm-year observations of Malaysian Shariah-compliant public listed companies across multiple industries. Variables included in the dataset comprise firm identification, industry classification, reporting year, reporting type, and disclosure scores for each of the thirteen integrated reporting components. An aggregated integrated reporting disclosure score was calculated by summing the component scores for each firm-year observation.

The dataset provides structured measures of integrated reporting disclosure practices within a Shariah-compliant corporate context. Researchers may use the dataset to examine corporate reporting transparency, integrated reporting adoption, and disclosure practices across firms and time periods. The dataset may also support comparative research on corporate reporting, sustainability disclosure measurement, and methodological applications of content analysis

Specifications Table

**Table:** 

Subject	Social Sciences
Specific subject area	Integrated reporting (IR) disclosure measurement and corporate reporting transparency
Type of data	Tables, Figures
Data collection	Corporate annual reports and integrated reports of Malaysian public listed companies for 2020–2023 were collected from company websites and Bursa Malaysia disclosures. Content analysis was conducted using a 13-component IR disclosure framework aligned with the International Integrated Reporting Council (IIRC) Framework. Each component was scored on a scale of 0–5 based on disclosure depth and quality. Firms that were not Shariah-compliant throughout the period and firms with incomplete reporting years were excluded. Data were coded and compiled using Microsoft Excel.
Data source location	Malaysia Bursa Malaysia Website: https://www.bursamalaysia.com/trade/trading_resources/listing_directory/main_market
Data accessibility	Repository name: Mendeley Data Data identification number: 10.17632/2kkwzhy8xm.2 Direct URL to data: https://data.mendeley.com/datasets/2kkwzhy8xm/1
Related research article	None

## 1 Value of the Data

• This dataset provides structured measures of IR disclosure for Malaysian Shariah-compliant public listed companies from 2020 to 2023 using a standardized 13-component scoring framework. The dataset converts qualitative corporate disclosures into quantified disclosure scores derived from publicly available corporate reports.
• The dataset allows researchers to examine IR disclosure practices across firms, industries, and reporting years. The structured scoring system enables comparative analysis of corporate reporting transparency within a Shariah-compliant corporate environment.
• The dataset may be reused to examine relationships between IR disclosure and variables such as corporate governance characteristics, financial performance, sustainability disclosure practices, and other firm-level attributes, facilitating further empirical research on corporate reporting quality and IR adoption.
• The coding framework and disclosure scoring structure can serve as a methodological reference for researchers conducting content analysis of corporate reporting disclosures in other countries or time periods.

## 2 Background

IR has emerged as an approach to corporate reporting that integrates financial and non-financial information to explain how organisations create value over time. The IIRC Framework outlines key content elements and guiding principles intended to enhance the connectivity and transparency of corporate disclosures [1]. As IR practices have developed, researchers have sought structured methods to evaluate the extent and quality of disclosures contained in corporate reports.

To facilitate empirical research on IR disclosure practices, a dataset was developed through systematic manual content analysis of corporate annual reports and integrated reports of Malaysian public listed companies for the period 2020–2023. The dataset applies a thirteen-component IR disclosure determination framework aligned with the IIRC Framework. Each component was evaluated using a standardized scoring scale to capture variations in disclosure practices across firm-year observations.

The dataset provides structured and coded disclosure information derived from publicly available corporate reports, enabling further research on IR disclosure measurement and corporate reporting practices.

Beyond providing a structured dataset, this study contributes a transparent and replicable coding framework for measuring integrated reporting disclosures. By operationalizing the content elements of the International Integrated Reporting Council into a systematic scoring instrument, the dataset offers a methodological reference for future research on corporate reporting, sustainability disclosure, and integrated reporting practices across different institutional contexts.

## 3 Data Description

The dataset contains IR disclosure scores for Malaysian Shariah-compliant public listed companies over the period 2020–2023. The dataset was constructed through manual content analysis of publicly available annual reports and integrated reports using a thirteen-component coding framework aligned with IIRC Framework. Each component is evaluated on a 0–5 ordinal scale, where higher scores indicate a higher level of disclosure quality and completeness.

Table 1 presents the distribution of sample firms by industry. The dataset consists of 94 Malaysian Shariah-compliant public listed companies observed over a four-year period from 2020 to 2023, resulting in 376 firm-year observations. The Consumer Products & Services industry represents the largest share of the sample with 20 companies (80 firm-year observations), followed by Property with 15 companies (60 observations), and both Construction and Industrial Products & Services with 10 companies each (40 observations respectively). Other industries in the sample include Technology, Energy, Plantation, Health Care, Utilities, Transportation & Logistics, Financial Services, and Telecommunications & Media.

**Table 1:** Industry distribution of sample firms and firm-year observations.

**INDUSTRY**	**Number of Year**	**Number of Company**	**Number of Observations**
CONSUMER PRODUCTS & SERVICES	4	20	80
PROPERTY	4	15	60
CONSTRUCTION	4	10	40
INDUSTRIAL PRODUCTS & SERVICES	4	10	40
TECHNOLOGY	4	8	32
ENERGY	4	7	28
PLANTATION	4	7	28
HEALTH CARE	4	5	20
UTILITIES	4	5	20
TRANSPORTATION & LOGISTICS	4	4	16
FINANCIAL SERVICES	4	2	8
TELECOMMUNICATIONS & MEDIA	4	1	4
**TOTAL**		**94**	**376**

Table 2 reports the mean scores of the thirteen IR components for each industry. The table also reports the total IR score (T_SCORE), which represents the aggregated disclosure score across all components.

**Table 2:** Mean scores of integrated reporting components by industry.

Component	Obs	VALUE	CAPITAL	SFO	GOV	BMOD
CONSTRUCTION	40	1.050	0.750	1.200	2.050	0.750
CONSUMER PRODUCTS & SERVICES	80	2.113	1.700	2.713	2.288	2.200
ENERGY	28	2.179	2.000	3.714	2.321	2.036
FINANCIAL SERVICES	8	3.750	3.500	4.000	2.250	3.750
HEALTH CARE	20	2.700	2.650	3.000	3.250	2.250
INDUSTRIAL PRODUCTS & SERVICES	40	3.200	3.250	3.375	3.575	3.100
PLANTATION	28	2.000	1.536	2.821	2.893	1.714
PROPERTY	60	1.617	1.633	1.333	2.433	1.400
TECHNOLOGY	32	0.844	0.906	2.156	1.688	0.938
TELECOMMUNICATIONS & MEDIA	4	5.000	5.000	5.000	5.000	5.000
TRANSPORTATION & LOGISTICS	16	3.750	3.750	4.125	3.563	4.188
UTILITIES	20	3.200	2.850	3.500	2.850	3.050

Component	Obs	RIOPP	PERF	OUTLOOK	STHR	CONT

CONSTRUCTION	40	1.575	1.650	1.100	2.575	1.175
CONSUMER PRODUCTS & SERVICES	80	2.625	2.513	2.338	3.275	2.600
ENERGY	28	3.571	3.643	3.071	2.964	3.357
FINANCIAL SERVICES	8	4.000	3.500	3.250	4.750	3.500
HEALTH CARE	20	2.650	3.100	3.000	3.800	3.100
INDUSTRIAL PRODUCTS & SERVICES	40	3.475	3.425	3.125	3.625	3.375
PLANTATION	28	3.250	3.143	2.429	2.679	2.607
PROPERTY	60	1.633	1.467	1.083	2.700	1.350
TECHNOLOGY	32	2.281	2.031	2.594	3.094	2.219
TELECOMMUNICATIONS & MEDIA	4	5.000	5.000	5.000	5.000	5.000
TRANSPORTATION & LOGISTICS	16	4.375	4.063	4.063	4.375	3.563
UTILITIES	20	3.000	3.050	3.200	4.000	3.250

Component	Obs	MATCONS	CUVAET	ENV	T_SCORE	

CONSTRUCTION	40	2.150	1.275	1.700	19.000	
CONSUMER PRODUCTS & SERVICES	80	3.350	3.125	3.550	34.388	
ENERGY	28	2.964	3.857	3.464	39.143	
FINANCIAL SERVICES	8	4.750	4.000	4.250	49.250	
HEALTH CARE	20	3.750	3.800	3.700	40.750	
INDUSTRIAL PRODUCTS & SERVICES	40	3.625	3.450	3.850	44.450	
PLANTATION	28	2.393	3.214	3.536	34.214	
PROPERTY	60	2.450	0.750	1.433	21.283	
TECHNOLOGY	32	3.469	2.688	3.000	27.906	
TELECOMMUNICATIONS & MEDIA	4	5.000	5.000	5.000	65.000	
TRANSPORTATION & LOGISTICS	16	4.438	4.375	4.750	53.375	
UTILITIES	20	3.750	3.600	4.150	43.450	

VALUE = Value Creation Process; CAPITAL = Capitals; SFO = Strategic Focus and KPIs; GOV = Governance; BMOD = Business Model; RIOPP = Risks and Opportunities; PERF = Performance; OUTLOOK = Future Orientation/Outlook; STHR = Stakeholder Relationships; CONT = Connectivity; MATCONS = Materiality and Conciseness; CUVAET = Culture, Values and Ethics; ENV = Environmental.

**Fig. 1:**
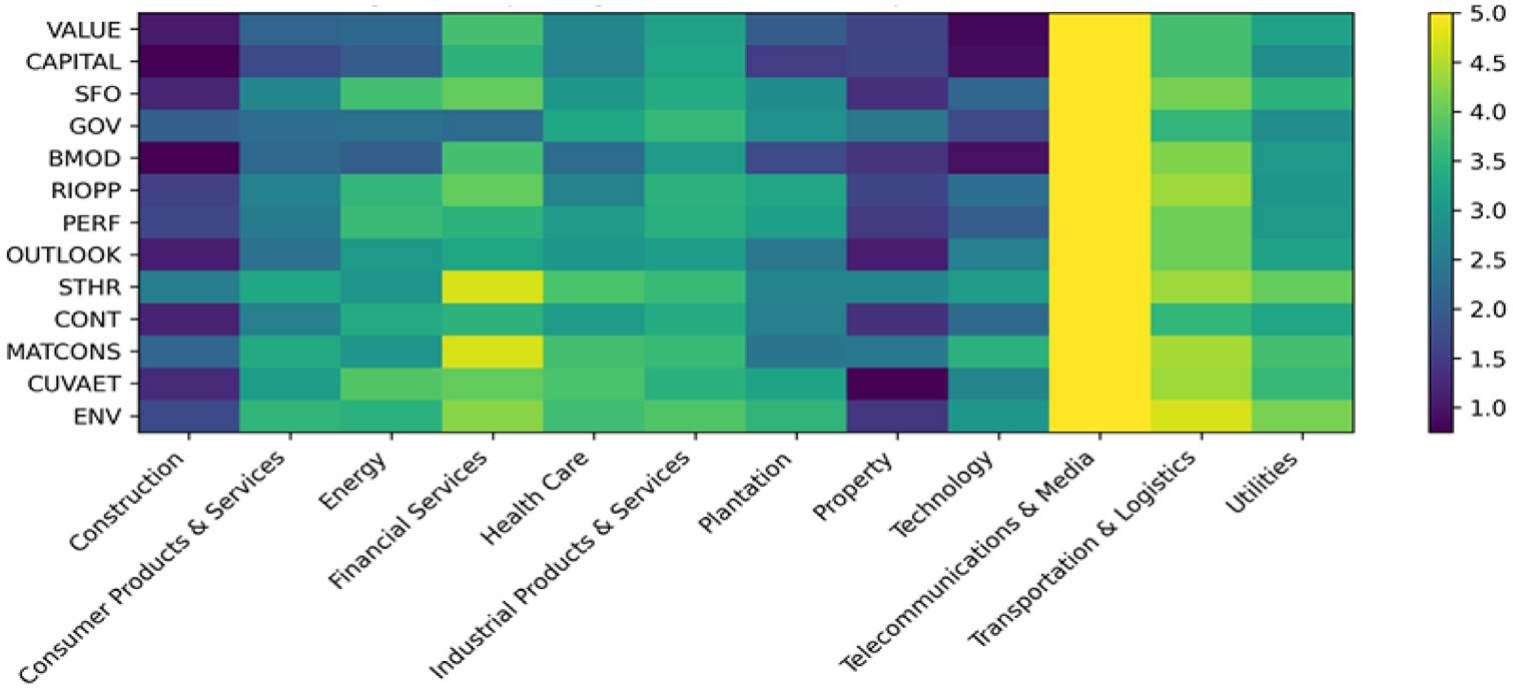
Integrated reporting disclosure landscape across industries.

Fig. 1 visualises the distribution of IR component scores across industries using a heatmap. The figure shows the relative intensity of disclosure across IR components and industries.

VALUE = Value Creation Process; CAPITAL = Capitals; SFO = Strategic Focus and KPIs; GOV = Governance; BMOD = Business Model; RIOPP = Risks and Opportunities; PERF = Performance; OUTLOOK = Future Orientation/Outlook; STHR = Stakeholder Relationships; CONT = Connectivity; MATCONS = Materiality and Conciseness; CUVAET = Culture, Values and Ethics; ENV = Environmental.

**Fig. 2:**
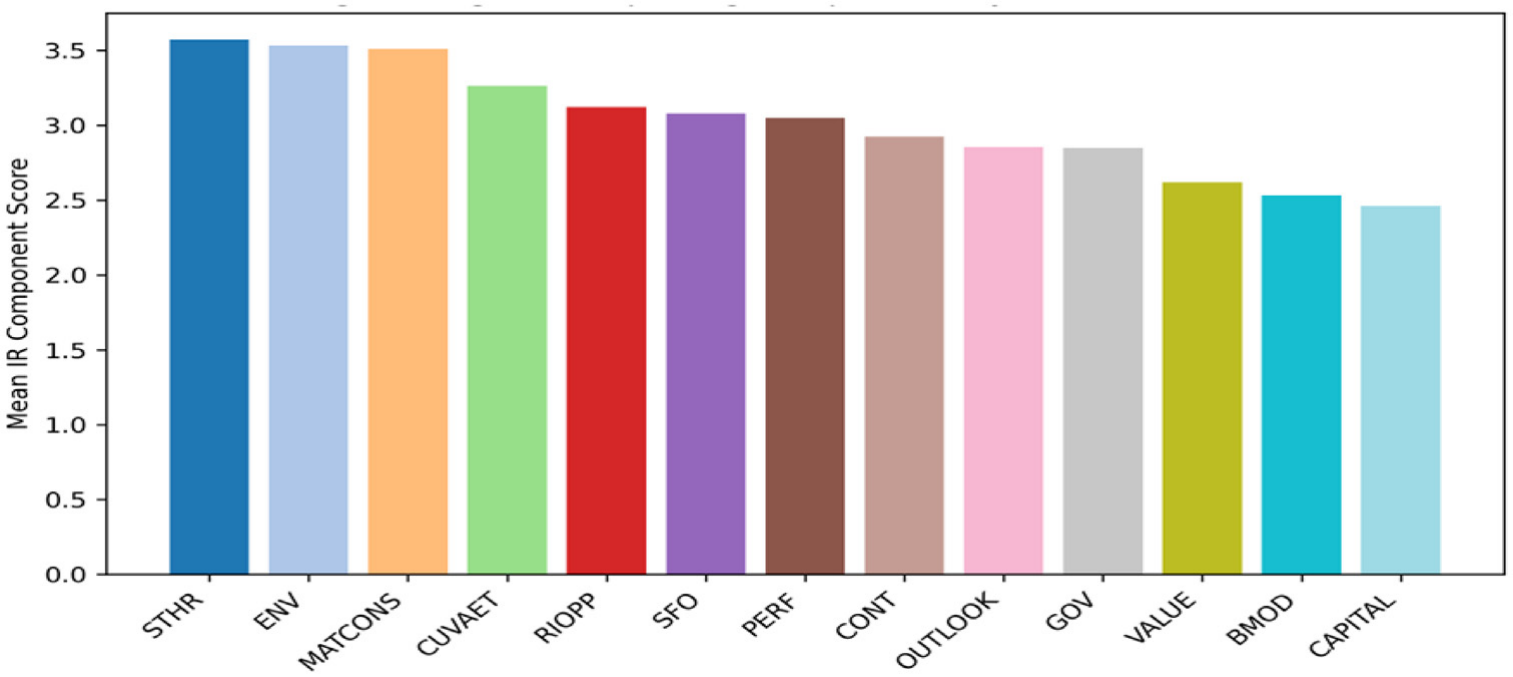
Ranking of integrated reporting components by mean disclosure score.

Fig. 2 presents the ranking of IR components based on their mean disclosure scores across the dataset.

VALUE = Value Creation Process; CAPITAL = Capitals; SFO = Strategic Focus and KPIs; GOV = Governance; BMOD = Business Model; RIOPP = Risks and Opportunities; PERF = Performance; OUTLOOK = Future Orientation/Outlook; STHR = Stakeholder Relationships; CONT = Connectivity; MATCONS = Materiality and Conciseness; CUVAET = Culture, Values and Ethics; ENV = Environmental.

The dataset supporting this study is publicly available in the Mendeley Data repository [2]. The raw disclosure scores used to construct the IR dataset are provided in the file “Table B_Integrated Reporting Disclosure Score.” The coding framework used for the content analysis, including the scoring criteria for each IR component, is documented in “Table A_Integrated Reporting (IR) Disclosure Determination Framework.” Table A presents the disclosure evaluation criteria, while Table B reports the firm-year IR disclosure scores generated from the coding process.

## 4 Experimental Design, Materials and Methods

The dataset was constructed through systematic manual content analysis of corporate reports of Malaysian public listed companies. The initial sampling frame consisted of the Top 200 Malaysian public listed companies ranked by market capitalization in 2020, which was used to identify large publicly listed firms with publicly available corporate reports. The year 2020 was used as the benchmark year for defining the sampling frame because it coincides with the onset of the COVID-19 pandemic, which prompted increased emphasis on corporate risk disclosure and transparency in financial reporting. Using the 2020 market capitalization ranking therefore provides a consistent and transparent benchmark for identifying large Malaysian public listed companies with publicly available corporate reports during this period of reporting adjustments [3,4].

From this initial sampling frame, companies that were not classified as Shariah-compliant throughout the period 2020–2023 were excluded. Shariah-compliant firms were identified based on the classification issued by the Shariah Advisory Council of the Securities Commission Malaysia [[5], [6], [7], [8]]. The Shariah screening methodology applies quantitative and qualitative criteria related to business activities and financial ratios to determine whether listed companies comply with Shariah investment principles [9]. Firms with incomplete corporate reporting data during the study period were also excluded to maintain a consistent panel of firm-year observations.

Corporate reports were collected for the period 2020–2023, including both annual reports and integrated reports. These reports were obtained from publicly accessible sources, including the Bursa Malaysia corporate disclosure portal and the official websites of the respective companies. For each firm-year observation, the report type was recorded to distinguish between annual reports and integrated reports.

The collected reports were systematically reviewed using a manual content analysis procedure. The analysis employed a thirteen-component IR disclosure determination framework following the approach adopted in prior studies [10], which is based on the IR disclosure principles developed in earlier literature [11] and the International Integrated Reporting Framework issued by IIRC [1]. The coding instrument evaluates disclosures relating to value creation process, capitals, strategic focus and KPIs, governance, business model, risks and opportunities, performance, future orientation/outlook, stakeholder relationships, connectivity, materiality and conciseness, culture, values and ethics, and environmental.

The thirteen codes capture different dimensions of integrated reporting disclosure. VALUE assesses how the organization explains its value creation process over the short, medium and long term. CAPITAL captures disclosure of the capital used or affected by the organization. SFO evaluates strategic focus, strategic priorities and the use of key performance indicators. GOV assesses disclosure on governance structure and its role in supporting value creation. BMOD evaluates the explanation of the business model, including inputs, business activities, outputs and outcomes. RIOPP captures disclosure of risks and opportunities and how they are managed. PERF assesses financial and non-financial performance outcomes. OUTLOOK evaluates forward-looking discussion of future prospects, challenges and uncertainties. STHR captures stakeholder identification, engagement and responsiveness. CONT evaluates connectivity of information across report sections. MATCONS assesses the disclosure of materiality and conciseness. CUVAET captures disclosures on culture, values and ethics. ENV evaluates environmental disclosures, including climate-related and environmental challenges linked to the business model or strategy.

Each disclosure component was evaluated using a 0–5 ordinal scoring scale, representing increasing levels of disclosure depth and quality. The scoring criteria were defined using a structured coding instrument describing the disclosure characteristics associated with each score level. During the coding process, the corporate reports were systematically examined to identify disclosures corresponding to each IR component, and scores were assigned based on the predefined coding criteria.

The coding was performed by two trained research assistants and reviewed by a supervision team consisting of two professors and two senior lecturers. Before full data collection commenced, the supervision team provided training to the research assistants on the application of the thirteen-component coding framework and the 0–5 scoring scale. A pilot coding exercise was conducted using one company report, during which the research assistants and supervision team coded the report together, compared the assigned scores, discussed differences in interpretation, and agreed on a common understanding of the scoring criteria.

Qualitative terms in the scoring scale, such as “limited”, “enhanced”, “clearly called out”, and “comprehensive”, were operationalized with reference to observable reporting characteristics. For example, disclosures were considered “clearly called out” when specific elements, such as capitals or value creation processes, were explicitly labelled and systematically explained in dedicated sections of the report. “Limited connectivity” refers to disclosures presented in isolation without cross-referencing across reporting elements, whereas “enhanced connectivity” reflects integrated narratives linking multiple elements, such as strategy, performance, risks, and capitals, within or across sections of the report. Similarly, “comprehensive disclosure” indicates detailed, structured, and internally consistent explanations supported by qualitative and/or quantitative information.

To further assess coding consistency, an inter-coder reliability check was conducted on approximately 10% of the firm-year observations. The same reports were independently coded by both research assistants, and the level of agreement was assessed using percentage agreement. The results indicated a high level of consistency in applying the coding framework. Any differences identified during this check were discussed with the supervision team, and clarifications were incorporated into the coding guidelines. This iterative calibration process strengthened the consistency and reproducibility of the coding across the full dataset.

During the full coding process, the research assistants collected and coded the disclosure data based on the established coding instrument. The coded data were subsequently reviewed by the supervision team. Any discrepancies or ambiguous scoring judgments were resolved through discussion between the research assistants and the supervision team until consensus was reached.

As part of ongoing efforts to further enhance the robustness and external validity of the coding framework, an independent validation exercise involving an external professional audit firm is currently in progress. This validation aims to assess the practical applicability and consistency of the scoring criteria from a practitioner perspective. As this process is ongoing, the results are not included in the present dataset. However, the current coding procedures, including training, pilot calibration, inter-coder reliability checking, and consensus-based review, provided a consistent and transparent basis for the dataset presented in this study.

The component-level scores were recorded for each firm-year observation and compiled into a structured dataset. The overall IR disclosure score was calculated by summing the scores across the thirteen disclosure components for each firm-year observation. The final dataset consists of 376 firm-year observations representing 94 Malaysian Shariah-compliant public listed companies for the period 2020–2023. All coding results were recorded and organized using Microsoft Excel, which was used to compile and structure the final dataset.

**Fig. 3:**
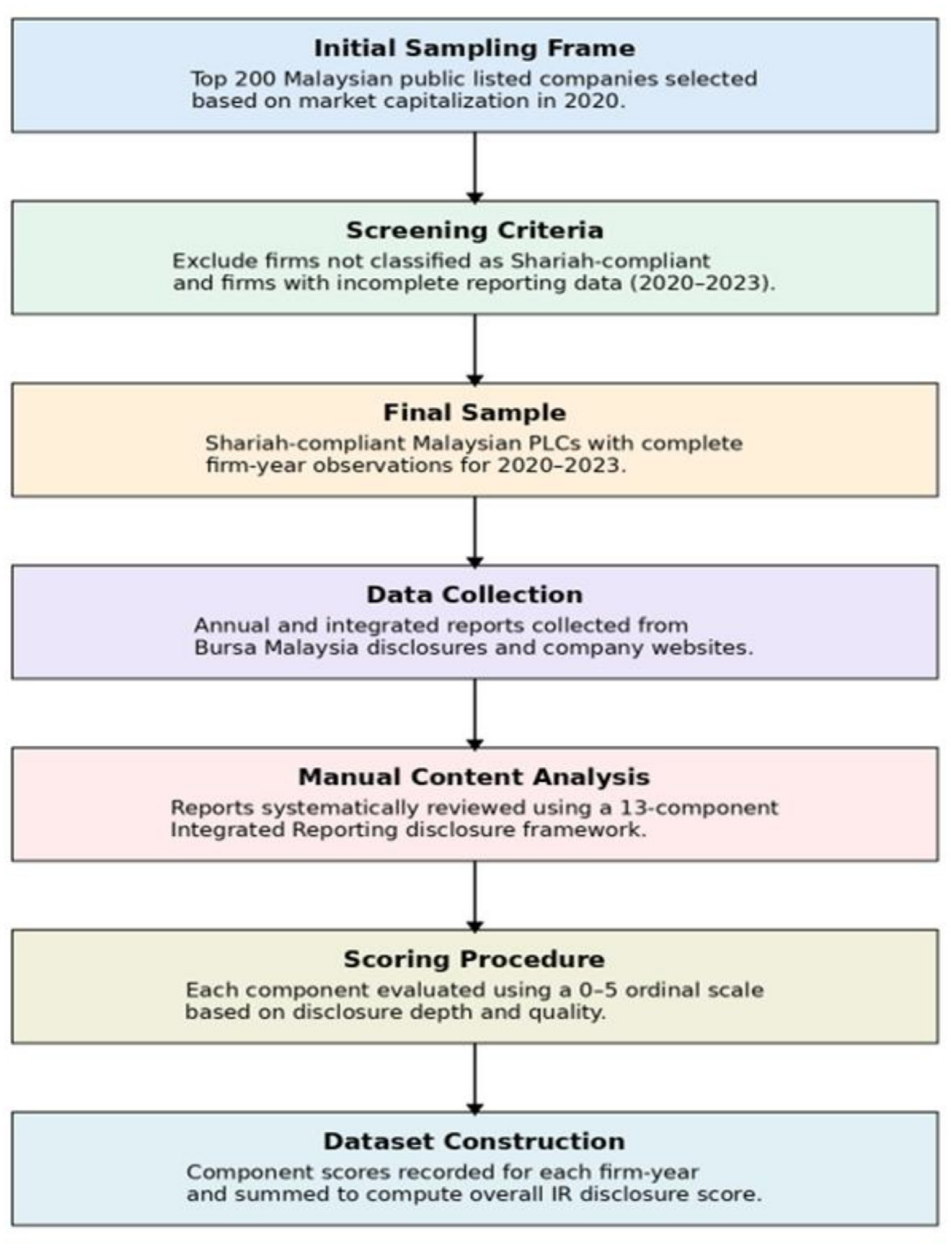
Data collection and dataset construction workflow for the integrated reporting disclosure dataset.

**Fig. 4:**
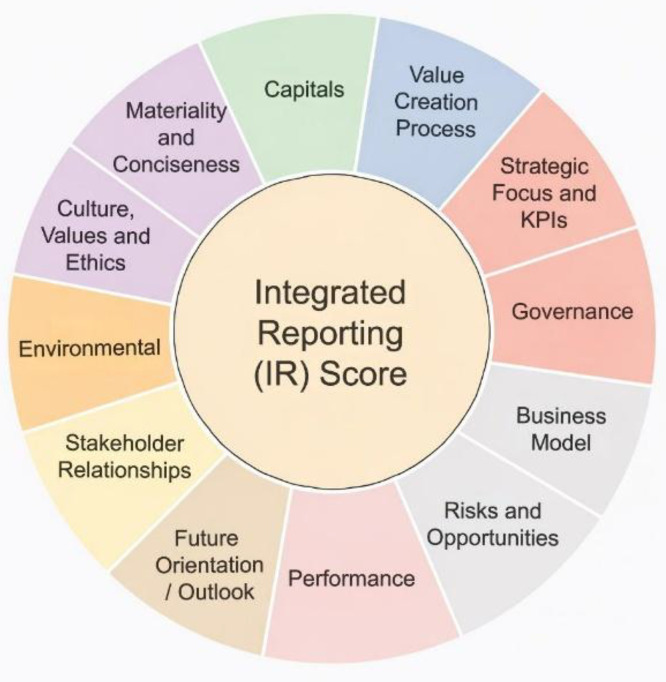
Integrated reporting disclosure scoring framework showing the thirteen components used to construct the IR disclosure score.

The overall data collection, screening, coding, and dataset construction process used to generate the dataset is illustrated in Fig. 3, while the IR disclosure scoring framework used in the coding process is presented in Fig. 4.

## Limitations

Not applicable.

## Ethics Statement

Throughout the data collection process, the authors adhered to all ethical considerations. The data used in this paper is not primary data, it is secondary data which is publicly available information from published reports. Therefore, the authors do not need to seek permission to use the secondary data. The authors also did not conduct human or animal experiments.

## Declaration of Generative AI and AI-assisted Technologies in the Manuscript Preparation Process

During the preparation of this manuscript, the authors used ChatGPT (OpenAI) to assist with language editing and improving the clarity of the manuscript text. The use of this tool was limited to language refinement. The authors carefully reviewed and edited the content after using this tool and take full responsibility for the accuracy and integrity of the final manuscript.

## CRediT authorship contribution statement

**Wai Kee Ho:** Conceptualization, Project administration, Supervision, Writing – original draft, Visualization. **Nampuna Dolok Gultom:** Methodology, Supervision. **Ferdinand Akthar Khan Gul:** Conceptualization, Methodology, Supervision. **Susela Devi K. Suppiah:** Validation, Supervision. **Khakan Najaf:** Conceptualization, Funding acquisition. **Emily Kok:** Investigation, Data curation. **Eng Sze Phuah:** Investigation, Data curation.

## Data Availability

Mendeley DataIntegrated Reporting Disclosure Determination and Scoring Dataset for Malaysian Shariah-Compliant Public Listed Companies (Original data)
Mendeley DataIntegrated Reporting Disclosure Determination and Scoring Dataset for Malaysian Shariah-Compliant Public Listed Companies (Original data)
